# Women’s emotional dependence on men and its relationship to intimate partner violence

**DOI:** 10.1192/j.eurpsy.2024.648

**Published:** 2024-08-27

**Authors:** F. Tabib, F. Guermazi, D. Mnif, F. Cherif, I. Feki, I. Baati, J. Masmoudi

**Affiliations:** ^1^Department of psychiatry A, UHC Hedi Chaker, Sfax, Tunisia

## Abstract

**Introduction:**

A woman’s emotional dependence on a man refers to a marked need for care, protection, and support, even in situations where the woman is able to function autonomously. This dependence fosters a fusional bond that makes it difficult for the woman to leave the relationship, however unhealthy it may be. This puts the victim at greater risk of suffering and tolerating violence, in particular intimate partner violence (IPV).

**Objectives:**

To study the emotional dependence of women who are victims of IPV, and to determine the factors associated with this dependence.

**Methods:**

We conducted a descriptive and analytical cross-sectional observational study, carried out over a 10-month period from March 2021 to December 2021, among female victims of IPV consulting psychiatric emergencies at UHC Hedi Chaker, Sfax, Tunisia for medical expertise at the request of the court.

Emotional dependence was assessed using the Emotional Dependence Questionnaire (EDQ) which contains 20 items. Responses are given on a seven-point Likert-type scale which is recoded so that a high score reflects a high level of emotional dependence in relationships.

**Results:**

The total number of participants was 120 with an average age of 37.27 years. The majority had secondary education or less (62.5%), were professionally active (53.3%), and were financially dependent on their partners (26.7%). As for the women’s clinical characteristics, 19.2% were under psychiatric care, 15% had attempted suicide and 10% had a history of childhood abuse. Tobacco was the only psychoactive substance consumed by 12.5% of the women. The average length of marriage was 12.34 years, exceeding 10 years in 44.2% of cases. Marital conflicts had existed since the very beginning of the relationship in 91.7% of cases.

The mean total score of the EDQ was 79.38, indicating a slight emotional dependence of these women on their spouses. It was correlated with childhood violence (p=0.028), smoking (p=0.049), early conflict (p<10-3), and personal psychiatric history (p=0.02).

**Conclusions:**

A link, probably bidirectional, may occur between emotional dependence and IPV, particularly a link with maintaining the relationship in an IPV context. However, the slight emotional dependence of our victims may explain why they seek help from the police and report the violence.

**Disclosure of Interest:**

None Declared

